# Autoimmune Thyroid Disease and Keratoconus: Is There an Association?

**DOI:** 10.1155/2018/7907512

**Published:** 2018-07-31

**Authors:** Hussam H. Alhawari, Yousef S. Khader, Hussein H. Alhawari, Amal F. Alomari, Hiba N. Abbasi, Muhannd S. El-Faouri, Muawyah D. Al Bdour

**Affiliations:** ^1^Division of Endocrinology and Metabolism, Department of Internal Medicine, School of Medicine, University of Jordan, Amman, Jordan; ^2^Department of Community Medicine, Public Health and Family Medicine, School of Medicine, Jordan University of Science and Technology, Irbid, Jordan; ^3^Department of Internal Medicine, School of Medicine, University of Jordan, Amman, Jordan; ^4^Division of Ophthalmology, Department of Special Surgery, School of Medicine, University of Jordan, Amman, Jordan

## Abstract

**Purpose:**

The association between autoimmune diseases and keratoconus (KC) has been proposed based on previous retrospective studies and case reports. The aim of our study is to investigate whether KC is associated with autoimmune thyroid disease*. Methods.* A comparative study was conducted on 131 adult subjects from September 2015 to May 2017 at Jordan University Hospital, Amman, Jordan. Subjects were classified into 2 groups: subjects with autoimmune thyroid disease, including Graves' disease and Hashimoto's thyroiditis (*n* = 68), and a healthy group for comparison (*n* = 63). Subjects with any other conditions known to be associated with KC were excluded. The diagnosis of KC was based on clinical and corneal topographic findings utilizing the Oculus-Pentacam machine. In addition, TSH and total T4 levels as well as thyroid peroxidase antibodies were measured in all study participants. Antithyroglobulin antibodies, thyroid stimulating immunoglobulin, thyroid ultrasound, and thyroid uptake and scan were also selectively performed in some participants.

**Results:**

This study included a total of 131 participants (101 females and 30 males), including patients and controls. In the multivariate analysis, autoimmune disease was not significantly associated with keratoconus (OR = 1.1; 95% confidence interval: 0.3, 3.8; *p* value = 0.353) after adjusting for age and gender.

**Conclusion:**

This study did not show a statistically significant association between autoimmune thyroid disease and KC.

## 1. Introduction

Keratoconus (KC) is a bilateral corneal ectasia characterized by central and paracentral corneal thinning, leading to visual impairment [1, 2]. KC has been generally classified as a noninflammatory disease, even though some reports suggest an underlying inflammatory process [3].

KC is typically a disease of younger people [4]. The incidence rate of KC among the general population is 1 per 50,000 [1]. Population studies in the Middle East have reported an incidence of KC of 20 per 100,000 in some areas of Saudi Arabia [5] and 24.9 per 100,000 in Iran [6]. A study performed in Lebanon, which screened medical students for KC, showed a prevalence of 3.3% [7]. Other studies carried out on the Arab population in the Palestinian Authority in Israel showed a prevalence of KC ranging from 1.5% to 3.0% [8–10]. The prevalence of KC in the general population may range anywhere from 0.05% to 0.22% depending on ethnicity and geographic location [11–13]. The higher prevalence of KC in the Middle East could be partly explained by the higher prevalence of vernal keratoconjunctivitis in this region [14, 15].

KC can be idiopathic or associated with an underlying etiology, including inheritance, environmental conditions, eye rubbing, contact lens use, or systemic disorders [16, 17].

The clinical presentation of KC can vary depending on disease severity. The early stages of KC usually do not produce any symptoms and can go unnoticed unless a screening test (i.e., corneal topography) is done; however, as the disease progresses, it can lead to a significant loss of visual acuity [1]. Even though there is no pharmacologic treatment currently available for KC, some recently introduced therapies, such as riboflavin and UV-A light, may alter the course and progression of the disease [18, 19]. Therefore, the routine screening of patients with risk factors for KC is essential for its early diagnosis and management.

The association between autoimmune diseases and KC has been suggested based on retrospective studies and case reports [20–24]. A recent study showed a prevalence of thyroid gland dysfunction of 13.6% in patients with KC [25].

Although other instances of thyroid-associated ophthalmopathy are well known [26, 27], no previous cross-sectional studies have been done to determine the association between autoimmune thyroid disease and KC. Therefore, the goal of this study is to determine the association between autoimmune thyroid disease and KC in Jordanian patients.

## 2. Materials and Methods

### 2.1. Subjects

This comparative study was conducted from September 2015 through May 2017 at the General Endocrinology Clinic of Jordan University Hospital (JUH), a tertiary medical center in Amman, Jordan. Of note, JUH is the main referral hospital for the Jordanian Ministry of Health; thus, it accepts patients from all over Jordan.

The study was approved by the Institutional Review Board (IRB) at Jordan University Hospital. Subjects with autoimmune thyroid disease including Graves' disease and Hashimoto's thyroiditis were recruited from the pool of patients who were attending the outpatient endocrinology clinic at the JUH during the study period. Healthy subjects without known personal or family history of thyroid disease or autoimmune diseases were studied as a control group and were selected from the pool of persons accompanying, but unrelated to, patients who attended the outpatient endocrinology clinic during the study. However, relatives of patients with other endocrine diseases were included. To help randomize subject selection for both groups, every third visitor to the endocrine clinic was invited to the study. In addition, and since the prevalence of autoimmune thyroid disease is higher in females than males [28], regarding the control group selection process, we selected 1 male for each 5 female controls.

The inclusion criteria for subjects in the case group were as follows: males or females above the age of 13, confirmed autoimmune thyroid disease based on positive autoimmune thyroid autoantibodies in serum (thyroid peroxidase antibodies, antithyroglobulin antibodies, or thyroid-stimulating immunoglobulin), positive thyroid ultrasonic findings for Hashimoto's thyroiditis [29] in the setting of primary or subclinical hypothyroidism (baseline TSH > 5.0 micro IU/ml, T4 < 19.05 pmol/l), or diffusely increased thyroid uptake and a scan consistent with Graves' disease in the setting of primary or subclinical hyperthyroidism (baseline TSH < 0.1 micro IU/ml, T4 > 9.0 pmol/l).

The inclusion criteria for controls were as follows: males or females above the age of 13; no personal or family history of KC, thyroid disease, or autoimmune diseases; normal laboratory tests for TSH and T4; and negative TPO antibodies.

The final analysis and diagnosis of KC was based on Pentacam tomographic results and classified according to the criteria listed in Table 1.

To eliminate known risk factors for developing KC, we excluded participants with a history of persistent eye rubbing, atopy, vernal keratoconjunctivitis (VKC), Down syndrome, Leber's congenital amaurosis, Turner's syndrome, or congenital rubella [14, 30–34].

After explaining the study's purpose and protocols, signed informed consent forms were obtained from all the screened subjects.

### 2.2. Assessments

Data on age and gender were recorded for each participant. Autoimmune thyroid disease status and information concerning the ingestion of any thyroid-related medications (e.g., thyroxine and antithyroid medications) were recorded for all patients. Four ml of blood was drawn by trained nurses, collected into plain collection tubes, and immediately sent to the JUH laboratory to perform the tests outlined below. Serum TSH and T4 were determined by means of a chemiluminescent microparticle immunoassay (CMIA). The normal reference ranges for TSH and T4 were determined to be 0.35–4.94 micro IU/ml and 9.01–19.05 pmol/l, respectively (Architect System, Abbott Ireland Diagnostics Division, Longford, Ireland; November 2015). Serum antithyroid peroxidase antibodies (anti-TPO) and antithyroglobulin antibodies (antithyroglobulin) were determined by means of a chemiluminescent microparticle immunoassay (CMIA). The normal reference ranges for anti-TPO and antithyroglobulin antibodies were determined to be less than 5.61 IU/ml and less than 2.0 IU/ml, respectively (Architect System, Abbott Park Diagnostics Division, Abbott Park, IL, USA; February 2015).

Thyroid ultrasound and thyroid uptake and scan studies were performed on an individual basis to confirm the diagnosis as needed [29].

The diagnosis of KC was based on clinical and corneal topographic findings utilizing the Oculus-Pentacam machine.

### 2.3. Statistical Analysis

Data were analyzed using the Statistical Package for the Social Sciences (IBM SPSS, version 20). Data were described using percentages and means (standard deviations). The differences in the demographic characteristics and the prevalence of keratoconus between those subjects with and without autoimmune disease were analyzed by means of the chi-square test. Binary logistic regression was used to analyze the association between autoimmune diseases and keratoconus after adjusting for gender and age. Although the study is age- and gender-matched, the effects of age and gender were adjusted because some patients had no control subject. A *p* value of less than 0.05 was considered statistically significant.

The sample size and power of the study were calculated using EpiCalc 2000. Assuming that the prevalence of KC in the general population is 7.0% and at alpha level of 0.05, our selected sample of 68 cases and 63 controls had a power of 80% to detect an odds ratio of 4.0 between autoimmune diseases and KC. A much larger sample size is needed to detect an odds ratio of 2.0; therefore, this study might be considered a pilot study.

## 3. Results

During the study period, a total of 141 subjects were screened, 134 of whom fulfilled the study's criteria. However, 3 subjects declined to proceed with the study after the initial screening, leaving 131 subjects (101 females and 30 males) to be enrolled. This includes 68 subjects with autoimmune thyroid disease and 63 gender- and age-matched healthy subjects in the control group. Fifty-five percent of the participants were below 40 years of age. Of the 131 subjects, 4 (3.1%) had KC and 7 (5.3%) were KC suspects.  Table 2 shows the participants' demographic and clinical characteristics according to autoimmune thyroid disease. Patients with or without autoimmune disease did not differ significantly in gender (*p* = 0.812) or age (*p* = 0.160). The prevalence of KC was 2.9% in patients with autoimmune thyroid disease and 3.2% in patients without autoimmune thyroid disease. There was no significant difference between the two groups in the prevalence of KC (*p* value = 0.958).

Table 3 shows the abnormal Pentacam results in KC patients with and without autoimmune thyroid. Participants labeled as KC (based on Pentacam tomographic criteria) from both control and case groups showed at least one of the following abnormal slit lamp findings: stromal thinning, anterior bulging of the cornea, Vogt's striae, Fleischer ring, and faint apical scar.

In the multivariate analysis, autoimmune disease was not found to be significantly associated with keratoconus (OR = 1.1; 95% confidence interval: 0.3, 3.8; *p* value = 0.353) after adjusting for age and gender (Table 4).

## 4. Discussion

Our study did not show a significant difference in the prevalence of KC and/or KC suspect between patients with or without autoimmune thyroid disease.

We compared our results with previous studies suggesting a possible association between autoimmune diseases and KC. The study by Nemet et al. [20] was a retrospective observational case-control study that evaluated 426 patients with KC for the presence of immune disorders, including Hashimoto's thyroiditis. These investigators found 22 cases of Hashimoto's thyroiditis. However, the control group subjects in this study were randomly selected and matched for age and gender without any clear exclusion criteria. Another recent report by Thanos et al. [25], published in 2016, was a prospective study in which they screened 154 patients with KC for thyroid gland dysfunction (TGD). These investigators found 21 patients with TGD (19 patients with hypothyroidism and 2 patients with hyperthyroidism) and a prevalence of 13.6%. However, these authors did not include a control group and only compared their results with data from the general population for the year 1977 [35]. On the other hand, when these authors measured thyroxine levels in tear fluid, they included a control group and showed that the thyroxine levels in the tear fluid of patients with KC were 2–50 times higher than those of subjects free of ocular pathology, regardless of their thyroid function. These results are consistent with those previously published by Kahán et al. in 1990 [36], suggesting that there is a localized thyroxine effect rather than a systemic one. A study by Stachon et al. suggested a higher free T4 concentration in the aqueous humor of KC patients [37]. In addition, other reports highlighted the importance of adequate thyroxine levels for corneal collagen synthesis [38, 39].

Interestingly, with regard to KC prevalence in the general population, our study results match previous studies conducted on Arab and Middle Eastern populations with a range in between 1.5% and 3.0% [9, 10]. A study by Shehadeh et al., carried out in 2015, included participants within the age group of 17–27 years of age and, based on Pentacam tomographic criteria, they reported a KC and KC suspect prevalence of 1.5% and 8.4%, respectively [9].

The limitations of our study were its relatively small sample size and the fact that we did not measure thyroxine levels in the tear fluid of the participants. This test is not available in our country.

The association between autoimmune disease and KC was not significant in our study, possibly due to the insufficient study power related to the small sample size. Prospective studies are needed to study this topic further, giving special attention to include patients with both immune and nonimmune thyroid disease, and further evaluating the benefits of testing tear fluid thyroxine levels in patients with KC.

## 5. Conclusion

Our study did not show a statistically significant association between autoimmune thyroid disease and KC. Until the results of additional prospective studies on this subject become available, we do not recommend the routine screening of patients with autoimmune thyroid disease for KC.

## Figures and Tables

**Table 1:** Diagnostic criteria of KC based on Pentacam tomography.

1	K-max reading >48 D
2	Superior-inferior difference on the 4.0 mm circle on the sagittal map >2.5 D
3	Inferior-superior difference on the 4.0 mm circle on the sagittal map >1.5 D
4	Superior-inferior corneal thickness difference on the 4.0 mm circle >30 *μ*m
5	Y-coordinate value of the thinnest location <−0.5 mm
6	Values >15 *μ*m within the central 4.0 mm on the anterior elevation map (best-fit sphere)
7	Values >20 *μ*m within the central 4.0 mm on the posterior elevation map (best-fit sphere)
8	Thinnest corneal thickness on the pachymetry map <470 *μ*m

KC: if 4 or more of the above criteria. KC suspect: if 2-3 of the above criteria. No KC (normal): if 0-1 of the above criteria. Note: whenever the participants had bilateral findings, the patient was labeled according to the worse eye.

**Table 2:** Participants' demographic and clinical characteristics according to autoimmune thyroid disease.

	Autoimmune thyroid disease		
	No *n* (%)	Yes *n* (%)	*p* value
*Gender*			0.812
Female	48 (76.2)	53 (77.9)	
Male	15 (23.8)	15 (22.1)	
*Age (years)*			0.160
<40	38 (60.3)	34 (50.0)	
40+	25 (39.7)	34 (50.0)	
*Keratoconus*			0.958
No	58 (92.1)	62 (91.2)	
Keratoconus	2 (3.2)	2 (2.9)	
Keratoconus suspect	3 (4.8)	4 (5.9)	

**Table 3:** Abnormal Pentacam results in KC patients with and without autoimmune thyroid disease.

	Abnormal Pentacam results in KC patients					
	With autoimmune thyroid disease			Without autoimmune thyroid disease		
	Mean	SD	Range	Mean	SD	Range
Thinnest corneal thickness (*μ*m)	512.7	35.1	461, 554	483.0	19.9	466, 506
Highest value on posterior elevation map (*μ*m)	28.7	14.4	8, 47	26.0	10.2	11, 37
Highest value on anterior elevation map (*μ*m)	10.0	4.1	6, 17	9.0	2.9	6, 13
Y-Coordinate (mm)	−0.4	0.3	−0.06, −0.85	−0.7	0.1	−0.55, −0.94
S-I Thickness map (*μ*m)	36.7	22.9	10, 75	27.0	11.2	10, 40
I-S Sagittal map (D)	2.3	1.5	1.3, 4	3.8	2.1	1.9, 5.7
S-I Sagittal map (D)	2.9	0.3	2.6, 3.2	2.8	^∗^	
K-Max (D)	49.0	2.8	46.3, 54.1	47.1	1.5	45.8, 49.1

KC: keratoconus; S-I: superior-inferior; I-S: inferior-superior. ^∗^Only single value is available.

**Table 4:** Multivariate analysis of the association between autoimmune thyroid disease and keratoconus (KC and KC suspect).

	OR	95% confidence interval		*p* value
Gender (male versus female)	2.8	0.8	11.2	0.124
Autoimmune thyroid disease (yes versus no)	1.1	0.3	3.8	0.397
Age (≥40 versus <40 years)	1.9	0.5	7.4	0.353

## Data Availability

The patient's demographics, laboratory, and corneal topography results used to support the findings of this study are available from the corresponding author upon request.
